# Book Notices

**Published:** 1869-01

**Authors:** 


### Book Notices.

The Opium Habit, with Suggestions as to the Remedy. New York: Harper & Brothers, Franklin Square. 1868.

This is a neatly-published volume of 334 pages. Its contents are as follows:—Introduction; A Successful Attempt to abandon Opium; De Quincey's "Confessions of an English Opium-Eater"; Opium Reminiscences of Coleridge; William Blair; Opium and Alcohol Compared; Insanity and Suicide from an Attempt to abandon Morphine; A Morphine Habit overcome; Robert Hall—John Randolph—William Wilberforce; What shall they do to be Saved? Outlines of the Opium Cure. From these headings, every reader will be able to judge concerning the contents of the book. It is well calculated to interest and instruct both non-professional and professional readers.

For sale by S. C. Griggs & Co., Chicago. Price, $1.50.

---

A Treatise on Physiology and Hygiene; for Schools, Families, and Colleges. By J. C. Dalton, M.D., Professor of Physiology in the College of Physicians and Surgeons of New York. With Illustrations. New York: Harper & Brothers, Publishers. 1868.

This is a small octavo volume of near 400 pages, printed on good type and paper, with many cuts as illustrations. The author has succeeded well in presenting the leading facts of physiology and hygiene, in a small compass, well arranged for study in schools and academies, and for reference by the general reader.

For sale by S. C. Griggs & Co., Chicago. Price, $1.75.

---

Lectures on the Study of Fever. By Alfred Hudson, M.D., M.R.C.A., Physician to the Meath Hospital. Philadelphia: Henry C. Lea. 1869. For sale by W. B. Keen & Co., Chicago.

This is a full-sized octavo volume of 316 pages. The publishers have done their part of the work well; and the author has given a very interesting discussion of the whole subject of fevers.

---

Annual Report of the Surgeon-General of U. S. Army for 1868.

We have received a copy of the Surgeon-General's Annual Report, and read it with interest and pleasure. Below, we copy the greater part of the report:—

At the date of my last Annual Report, epidemic cholera and yellow fever prevailed among the troops in various sections of the country, a very full and exhaustive report of which was published for the information of medical officers of the army, in Circular No. 1, War Department, Surgeon-General's Office, June 10, 1868. To this date, there has been no well-authenticated case of epidemic cholera or yellow fever reported as occurring among troops in the present year.

The Monthly Reports of sick and wounded, for the fiscal year terminating June 30, 1868, received in the Division of Records of this Office to this date, represent an average mean strength of forty-five thousand two hundred and fifty-seven (45,257) white, and four thousand seven hundred and seventy-four (4,774) colored, troops.

For the *white troops*, the total number of cases of all kinds reported under treatment was one hundred and thirty-one thousand five hundred and eighty-one (131,581), or two thousand nine hundred and eight (2,908) per thousand (1,000) of strength —nearly three entries on the sick report, during the year, for each man. Of this number of cases, one hundred and eighteen thousand nine hundred and twenty-five (118,925) were for disease alone, and twelve thousand six hundred and fifty-six (12,656) for wounds, accidents, and injuries; being two thousand six hundred and twenty-eight (2,628) per thousand (1,000) of strength for disease, and two hundred and eighty (280) per thousand (1,000) of strength for wounds, accidents, and injuries. The average number constantly on sick report was two thousand eight hundred and fifty-two (2,852), of whom two thousand five hundred and ten (2,510) were sick and three hundred and forty-two (342) wounded, or fifty-five (55) per thousand (1,000) constantly under treatment for disease, and eight (8) per thousand (1,000) for wounds and injuries. The total number of deaths from all causes reported, was one thousand three hundred and fifty-three (1,353); of which, one thousand one hundred and seventy-five (1,175) were from disease, and one hundred and seventy-eight (178) for wounds, accidents, and injuries; being at the rate of twenty-six (26) deaths from disease and four (4) from wounds, to each thousand (1,000) of strength. Of the deaths from disease, four hundred and twenty-seven (427) were from yellow fever, one hundred and thirty-nine (139) from cholera, and six hundred and nine (609), or thirteen (13) deaths per thousand (1,000) of strength, from all other diseases. The proportion of deaths from all causes to cases treated, was one (1) death to ninety-seven (97) cases.

Nine hundred and eighty-four (984) white soldiers, or twenty-two (22) per thousand (1,000) of strength, were discharged upon Surgeon's certificate of disability.

For the *colored troops*, the whole number of cases, of all kinds, treated, was fourteen thousand six hundred and sixteen (14,616); being at the rate of three thousand and sixty-one (3,061) per thousand (1,000) of strength, or three (3) cases of sickness for each man. Of this number, thirteen thousand five hundred and fifty (13,550) were for disease; being two thousand eight hundred and thirty-eight (2,838) per thousand (1,000) of strength; one thousand and sixty-six (1,066) were for wounds, accidents, and injuries; being two hundred and twenty-three (223) per thousand (1,000). The average number constantly on sick report was two hundred and eighty-three (283); of whom two hundred and forty-eight (248) were sick, and thirty-five (35) wounded; being at the rate of fifty-two (52) per thousand (1,000) constantly under treatment for disease, and seven (7) per thousand (1,000) for wounds, accidents, and injuries.

The total number of deaths reported was two hundred and sixty-eight (268); of which, two hundred and forty-two (242) were from disease, twenty-six (26) from wounds and injuries; being at the rate of fifty-one (51) deaths per thousand (1,000) of strength from disease, and five (5) per thousand (1,000) from wounds. Of the deaths from disease, twenty-five (25) were from yellow fever, eighty-nine (89) from cholera; leaving one hundred and twenty-eight (128), or twenty-seven (27) per thousand (1,000) of strength, from all other diseases. The proportion of deaths from all causes to cases treated, was one (1) death to fifty-five (55) cases.

Ninety (90) colored soldiers, or nineteen (19) per thousand (1,000) of strength, were discharged on Surgeon's certificate of disability.

During the year, the records filed in the Record and Pension

Division of this Office have been searched, and such official information relative to deaths, discharges, and treatment as they contain has been furnished, in reply to the inquiries of the Pension Bureau, in 16,786 cases; Adjutant-General, U.S.A., in 15,582 cases; Paymaster-General, U.S.A., in 473 cases; and in 1,929 cases to other authorized inquirers; making a total of 34,770.

In the Division of Surgical Records, the histories of 74,954 cases of wounds and injuries have been transcribed, chiefly from field reports, hospital case-books, and registers of 1861 and 1862, and the earlier part of 1863.

The records of the Office, in regard to injuries of the head, face, neck, thorax, abdomen, spine, and pelvis, have been classified and studied. Illustrative cases have been selected and written out in minute detail, while numerical tables have been prepared, exhibiting the progress and results of the different classes of injuries to which these individual examples belong. To illustrate these injuries, for future publication, there have been completed, during the year, 8 chromo-lithographs, 8 lithographs, and 3 diagrams. There have also been prepared, during the year, 122 woodcuts, to be intercalated in the text, descriptive of the various classes of injuries and operations. 500 pages of manuscript are in readiness for the printer, and a large amount of the statistical material is in such a state of forwardness that it can be made ready for the press at a few weeks' notice. To make the publications of this Office as valuable as possible, in relation to the results of the major surgical injuries and operations, and especially in regard to the excisions of the larger joints, and other operations embraced under the general designation of conservative surgery, much time and labor have been expended in tracing the ultimate histories of patients who have undergone such mutilations. This has been accomplished to a very satisfactory degree, through the coöperation of the examining surgeons of the Pension Bureau, of the Surgeons-General and Adjutants-General of the several States, of retired volunteer medical officers, and of private physicians. Besides the digestion and tabulation of the surgical data pertaining to the late war, there have been received and consolidated, 699 quarterly reports of post-hospitals, 34 reports of the examination of men, who, having been wounded, presented themselves for reënlistment at recruiting stations, and 32 special reports of surgical operations.

The Army Medical Museum continues to increase in value and usefulness. During the year, 673 specimens have been added to the surgical section, 121 to the medical section, 202 to the section of comparative anatomy, 687 specimens and 114 photographic negatives of microscopical specimens to the microscopical section. An anatomical section, of 163 specimens, has been formed, and is rendered of especial interest by the large proportion of typical crania of the North American aborigines which it contains. A collection of 187 specimens of Indian weapons and utensils has also been added. 266 discarded specimens, the histories of which could not be found at the period of publication of the catalogue of the surgical section, have been identified and restored to the collection. For purposes of exchange with other museums, or with learned societies, either for specimens or publications, 4,472 photographs, illustrative of injuries and operations, have been printed. There were, during the year, 14,448 visitors to the Museum, including many military surgeons of eminence.

On the 30th of September, there were 289 garrisoned posts in the various Military Departments, besides an almost equal number of detachments on temporary duty, throughout the South, and on expeditions, or protecting the lines of travel on the plains, requiring medical attendance. The number of surgeons and assistant-surgeons being altogether inadequate to meet this demand, it has been necessary to employ contract-physicians, especially at the South, where but few of the resident physicians could take the oath necessary to their payment, and the fees for attendance in individual cases would be far in excess of the contract rates. The number of physicians so employed upon the 30th of September was 282, at rates of compensation varying from $45.00 to $125.00 per month; but a large proportion of these will be dispensed with so soon as the troops are concentrated in winter-quarters, and the condition of public affairs will admit of the discontinuance of the numerous small garrisons throughout the States recently in rebellion.

Since the date of my last Annual Report, 3 surgeons and 2 assistant-surgeons have died, 8 assistant-surgeons have resigned, 2 assistant-surgeons have been dismissed, and 1 assistant-surgeon cashiered—total, 16.

A medical board, for the examination of candidates for appointment as assistant-surgeons, U.S. Army, and of assistant-surgeons for promotion, is now in session in New York City.

There are now 49 vacancies in the grade of Assistant-Surgeon. Most respectfully, your obedient servant,

J. K. BARNES,

*Surgeon-General, U.S. Army.*

